# Disparate Effects of Stilbenoid Polyphenols on Hypertrophic Cardiomyocytes In Vitro vs. in the Spontaneously Hypertensive Heart Failure Rat

**DOI:** 10.3390/molecules22020204

**Published:** 2017-02-01

**Authors:** Bolanle C. Akinwumi, Pema Raj, Danielle I. Lee, Crystal Acosta, Liping Yu, Samuel M. Thomas, Kalyanam Nagabhushanam, Muhammed Majeed, Neal M. Davies, Thomas Netticadan, Hope D. Anderson

**Affiliations:** 1 College of Pharmacy, Rady Faculty of Health Sciences, University of Manitoba, 750 McDermot Avenue, Winnipeg, MB R3E 0T5, Canada; bakinwumi@sbrc.ca (B.C.A.); dlee@sbrc.ca (D.I.L.); ndavies@ualberta.ca (N.M.D.); 2 Canadian Centre for Agri-Food Research in Health and Medicine, St. Boniface Hospital Research Centre, 351 Taché Avenue, Winnipeg, MB R2H 2A6, Canada; praj@sbrc.ca (P.R.); cacosta@sbrc.ca (C.A.); lyu@sbrc.ca (L.Y.); 3 Department of Physiology and Pathophysiology, Max Rady College of Medicine, University of Manitoba, 753 McDermot Avenue, Winnipeg, MB R3E 0T6, Canada; 4 Agriculture and Agri-Food Canada, St. Boniface Hospital Research Centre, 351 Taché Avenue, Winnipeg, MB R2H 2A6, Canada; 5 Department of Pharmacology and Therapeutics, Max Rady College of Medicine, University of Manitoba, 753 McDermot Avenue, Winnipeg, MB R3E 0T6, Canada; 6 Sami Labs Ltd., Peenya Industrial Area, Bangalore 560058, India; samuelmanoharan@samilabs.com (S.M.T.); mmajeed@sabinsa.com (M.M.); 7 Sabinsa Corporation, 20 Lake Drive, East Windsor, NJ 08520, USA; kalyanam@sabinsa.com; 8 Faculty of Pharmacy and Pharmaceutical Sciences, University of Alberta, 2-35, Medical Sciences Building, Edmonton, AL T6G 2H7, Canada

**Keywords:** hypertension, heart failure, resveratrol, polyphenol, stilbenoid

## Abstract

Stilbenoids are bioactive polyphenols, and resveratrol (*trans*-3,5,4′-trihydroxystilbene) is a representative stilbenoid that reportedly exerts cardioprotective actions. As resveratrol exhibits low oral bioavailability, we turned our attention to other stilbenoid compounds with a history of medicinal use and/or improved bioavailability. We determined the effects of gnetol (*trans*-3,5,2′,6′-tetrahydroxystilbene) and pterostilbene (*trans*-3,5-dimethoxy-4′-hydroxystilbene) on cardiac hypertrophy. In vitro, gnetol and pterostilbene prevented endothelin-1-induced indicators of cardiomyocyte hypertrophy including cell enlargement and protein synthesis. Gnetol and pterostilbene stimulated AMP-activated protein kinase (AMPK), and inhibition of AMPK, using compound C or shRNA knockdown, abolished these anti-hypertrophic effects. In contrast, resveratrol, gnetol, nor pterostilbene reduced blood pressure or hypertrophy in the spontaneously hypertensive heart failure (SHHF) rat. In fact, AMPK levels were similar between Sprague-Dawley and SHHF rats whether treated by stilbenoids or not. These data suggest that the anti-hypertrophic actions of resveratrol (and other stilbenoids?) do not extend to the SHHF rat, which models heart failure superimposed on hypertension. Notably, SHHF rat hearts exhibited prolonged isovolumic relaxation time (an indicator of diastolic dysfunction), and this was improved by stilbenoid treatment. In conclusion, stilbenoid-based treatment as a viable strategy to prevent pathological cardiac hypertrophy, a major risk factor for heart failure, may be context-dependent and requires further study.

## 1. Introduction

Cardiac hypertrophy refers to the increased myocardial mass provoked by hemodynamic stress or myocardial injury [1], and is a convergence point for heart failure risk factors. Prolonged hypertrophy leads to functional decompensation [2,3,4], so mitigation of this process is considered a promising therapeutic target to prevent heart failure [5].

Stilbenoids are a family of bioactive polyphenols, and resveratrol (*trans*-3,5,4′-trihydroxystilbene) is a representative stilbenoid that has been linked to improved longevity, cardiovascular, and neurodegenerative health [6,7,8,9]. In fact, we reported the effects of resveratrol on the microvasculature and heart in the spontaneously hypertensive rat (SHR), an experimental model of hypertension and cardiac hypertrophy [10,11]. However, although resveratrol is well-tolerated in humans, it is poorly soluble and readily metabolized. Rapid glucuronidation during phase II conjugation results in low oral bioavailability (~20%) and a half-life of ~14 min [12,13,14].

There are other stilbenoid compounds with therapeutic potential. Pterostilbene (*trans*-3,5-dimethoxy-4′-hydroxystilbene) is a dimethylated analog of resveratrol found in grapes [15] and blueberries [16], and is used in Ayurvedic medicine to treat coronary heart disease [17]. As the 3 position is methylated, it is protected from glucuronidation. Thus, resveratrol is a much better substrate for the UDG-glucuronosyltransferase family of enzymes, and it is more readily glucuronidated than pterostilbene. In fact, pterostilbene exhibits 95% oral bioavailability and a half-life of 105 min [14,18]. Gnetol (*trans*-3,5,2′,6′-tetrahydroxystilbene) is a structural analog of resveratrol from the genus *Gnetum* [19,20,21,22]. In Southeast Asia, seeds and fruit of *G. gnemon*, commonly called melinjo, are consumed as traditional foods [23]. Melinjo seed extracts and gnetol are used in Asian traditional medicine [22,23], and *Gnetum* extracts are widely used as natural health products [22,24].

Given the importance of cardiac hypertrophy as a risk factor for heart failure, and based on previous reports that resveratrol suppresses hypertrophy [11,25,26,27], we queried whether pterostilbene, and perhaps gnetol, would produce greater cardioprotective effects. We began by characterizing the ability of pterostilbene and gnetol to suppress cardiomyocyte hypertrophy in vitro, as previously reported for resveratrol [28,29,30,31].

We also probed AMP-activated protein kinase (AMPK) as a potential mediator of stilbenoid effects. AMPK is a serine/threonine kinase that acts as a cellular energy sensor. In cardiomyocytes, AMPK modulates energy homeostasis by: (i) increasing fatty acid uptake and oxidation; (ii) accelerating glucose uptake; (iii) stimulating glycolysis; and (iv) attenuating energy-consuming pathways such as protein synthesis [32]. In fact, activated AMPK exerts anti-hypertrophic effects such as blockade of cardiomyocyte enlargement, protein synthesis, hypertrophic gene expression, and pro-hypertrophic signaling in vitro [33,34,35,36] as well as in vivo [36], and the ability of resveratrol [29] to impede hypertrophy has been attributed to AMPK signaling. Finally, we carried out a comparative study of the three stilbenoid polyphenols in vivo.

## 2. Results

### 2.1. Effects of Gnetol and Pterostilbene on Cardiomyocyte Hypertrophy and Viability

**Figure 1:**
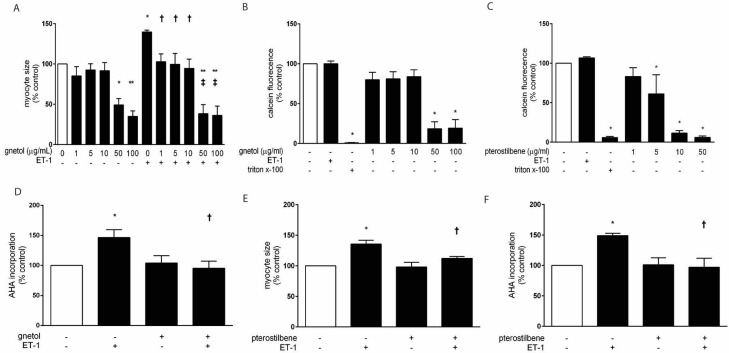
Effects of gnetol and pterostilbene on cardiomyocyte hypertrophy and viability. (**A**) The ability of ET1 (0.1 µM; 24 h) to induce myocyte enlargement was abolished by lower concentrations of gnetol (1–10 µg/mL), whereas higher concentrations of gnetol (50–100 µg/mL) reduced cell size in the presence and absence of ET1. *n* = 3; 40–45 myocytes/group. * *p* < 0.05 and ** *p* < 0.01 vs. control (open bars); ^†^ *p* < 0.05 and ^‡^ *p* < 0.01 vs. ET1. The effects of gnetol and pterostilbene on cardiomyocyte viability were therefore determined using triton x-100 as a positive control of reduced cardiomyocyte viability; (**B**) Lower concentrations of gnetol (1, 5, and 10 µg/mL) exhibited no adverse effects on calcein fluorescence (an indicator of viable cardiomyocytes), whereas higher concentrations (50 and 100 µg/mL significantly decreased viability. *n* = 3–4. * *p* < 0.05 vs. control (open bars); (**C**) only 1 µg/mL of pterostilbene exhibited no adverse effects on viability, whereas higher concentrations (5, 10 and 50 µg/mL) significantly decreased calcein fluorescence. *n* = 3–4. * *p* < 0.05 vs. control (open bars); (**D**) A sub-maximal concentration of gnetol (5 µg/mL) blocked ET1-induced protein synthesis (measured as l-azidohomoalanine [AHA] incorporation), a second marker of hypertrophy. *n* = 3; * *p* < 0.05 vs. control (open bars); ^†^ *p* < 0.05 vs. ET1. The ability of ET1 (0.1 µM; 24 h) to induce; (**E**) myocyte enlargement and (**F**) protein synthesis (i.e., AHA incorporation) was abolished by pterostilbene (1 µg/mL). *n* = 3; 40–45 myocytes/group. * *p* < 0.05 vs. control (open bars); ^†^ *p* < 0.05 vs. ET1.

We previously reported that ~7 µg/mL of resveratrol was required to attenuate norepinephrine-induced hypertrophy of cardiac myocytes [31]. Therefore, we confirmed the anti-hypertrophic actions of resveratrol in ET1-treated myocytes (Supplementary Figure S1). We then began by assessing the effect of increasing concentrations of gnetol within a similar range (0–100 µg/mL) on hypertrophic growth. ET1 treatment (0.1 µM; 24 h) elicited hypertrophy, as evidenced by significant enlargement of myocytes (Figure 1A). Lower concentrations of gnetol (1–10 µg/mL) abolished ET1-induced myocyte enlargement, but did not affect untreated myocytes. In contrast, higher concentrations of gnetol (50–100 µg/mL) markedly reduced cell size in the presence and absence of ET1, which suggests toxicity rather than anti-growth effects.

Having detected possible toxic effects of higher-concentration gnetol, we next measured the effects of increasing concentrations of gnetol (1–100 µg/mL) and pterostilbene (1–50 µg/mL) on cardiomyocyte viability. We confirmed that lower concentrations of gnetol (1, 5, and 10 µg/mL) and pterostilbene (1 µg/mL) exhibited no adverse effects on calcein fluorescence, whereas higher concentrations (gnetol: 50 and 100 µg/mL; pterostilbene: 5, 10 and 50 µg/mL) significantly decreased viability (Figure 1B,C, respectively). Based on these data, 5 µg/mL and 1 µg/mL were selected as the working concentrations of gnetol and pterostilbene, respectively. At these concentrations, gnetol also blocked ET1-induced protein synthesis (Figure 1D), a second marker of hypertrophy, and pterostilbene likewise attenuated ET1-induced myocyte enlargement and protein synthesis (Figure 1E,F). These data suggest that gnetol and pterostilbene exhibit anti-hypertrophic properties in isolated cardiac myocytes.

### 2.2. AMPK Mediates the Anti-Hypertrophic Effects of Pterostilbene and Gnetol

**Figure 2:**
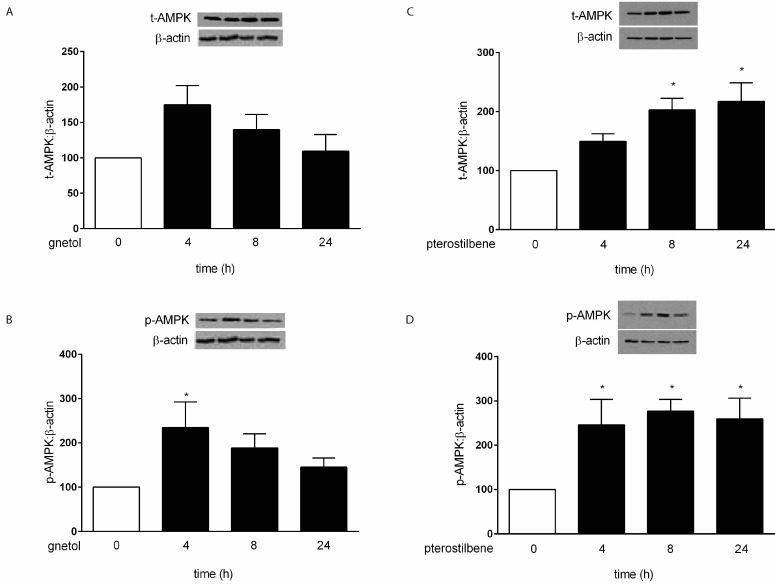
Gnetol and pterostilbene increase AMPKα levels and/or phosphorylation. Gnetol (5 µg/mL) (**A**) did not significantly affect total AMPK levels, and yet (**B**) promoted activation of AMPKα as reflected by phosphorylation at Thr172. Pterostilbene (1 µg/mL) increased both (**C**) total AMPK levels and (**D**) AMPKα phosphorylation. *n* = 3–4. * *p* < 0.05 vs. control (open bars).

**Figure 3:**
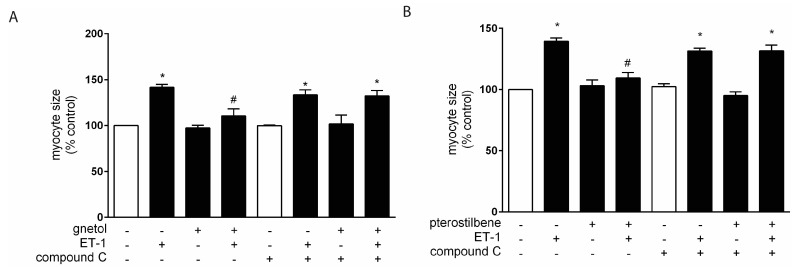
Chemical disruption of AMPK signaling attenuates gnetol and pterostilbene effects. Myocytes pre-treated with vehicle or compound C, a chemical inhibitor of AMPK, for 1 h, then exposed to gnetol or pterostilbene for 1 h, followed by addition of ET-1 (0.1 µM) for 24 h. Upon addition, all compounds remained in the culture media for the remainder of the experiment. Compound C abolished the ability of (**A**) gnetol (5 µg/mL); and (**B**) pterostilbene (1 µg/mL) to inhibit ET1-induced myocyte enlargement. *n* = 3. * *p* < 0.05 vs. control (open bars); ^#^ *p* < 0.05 vs. ET1.

**Figure 4:**
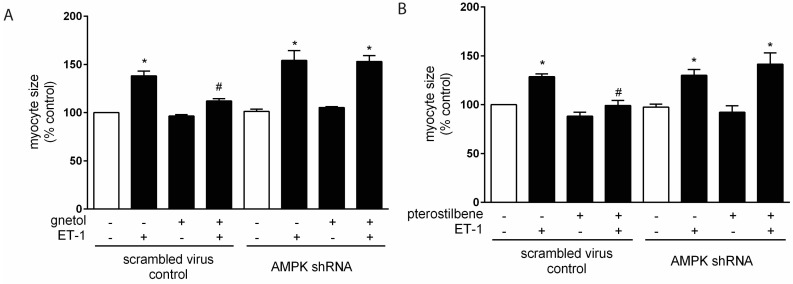
shRNA knockdown AMPKα abolishes gnetol and pterostilbene effects. Following simultaneous knockdown of AMPKα_1_ and AMPKα_2_, the catalytic subunits of AMPK, myocytes were exposed to gnetol or pterostilbene for 1 h, followed by addition of ET-1 (0.1 µM) for 24 h. Upon addition, all compounds remained in the culture media for the remainder of the experiment. AMPKα knockdown abrogated the ability of (**A**) gnetol (5 µg/mL); and (**B**) pterostilbene (1 µg/mL) to abolish ET1-induced myocyte enlargement. *n* = 3. * *p* <0.05 vs. control (open bars); ^#^ *p* < 0.05 vs. ET1.

As discussed above, we identified AMPK as a candidate mediator of pterostilbene and gnetol effects. Levels of total AMPK were not affected by gnetol (Figure 2A), though we observed significantly increased phosphorylation of AMPKα at Thr172, which is an indicator of AMPK activation status [37,38] (Figure 2B). Total levels of AMPK as well as phosphorylation of AMPKα at Thr172 were increased by pterostilbene (Figure 2C, D). We next disrupted AMPK signaling chemically using compound C [6-[4-(2-piperidin-1-ylethoxy)phenyl]-3-pyridin-4-ylpyrazolo[1,5-a]pyrimidine]; 1 µM) or by shRNA knockdown of AMPKα_1/2_. Infection of cardiomyocytes with lentiviral constructs expressing shRNA against AMPKα_1_ and AMPKα_2_ produced significant, simultaneous reductions to 29% ± 4% and 39% ± 14%, respectively (*n* = 3, *p* < 0.05). Compound C (Figure 3) and AMPKα_1/2_ knockdown (Figure 4) abolished the ability of gnetol and pterostilbene to attenuate cardiomyocyte hypertrophy, suggesting that AMPK is a key player.

### 2.3. In Vivo Effects of Resveratrol, Gnetol, and Pterostilbene on Blood Pressure, Cardiac Structure and Cardiac Function

**Figure 5:**
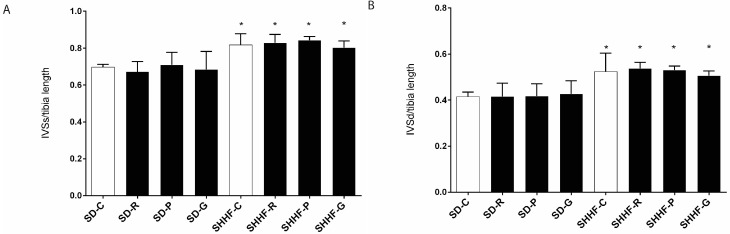
Stilbenoid treatment does not attenuate cardiac hypertrophy in the SHHF rat. Normalized (**A**) IVSs and (**B**) IVSd were increased in untreated SHHF (SHHF-C) compared to untreated SD (SD-C) rat hearts. 8-week treatment with resveratrol (R; 2.5 mg/kg/day), pterostilbene (P; 2.5 mg/kg/day), or gnetol (G; 2.5 mg/kg/day) did not affect normalized (**A**) IVSs and (**B**) IVSd in hearts from SD nor SHHF rats. *n* = 6–8. * *p <* 0.05 vs. SD-C hearts.

**Figure 6:**
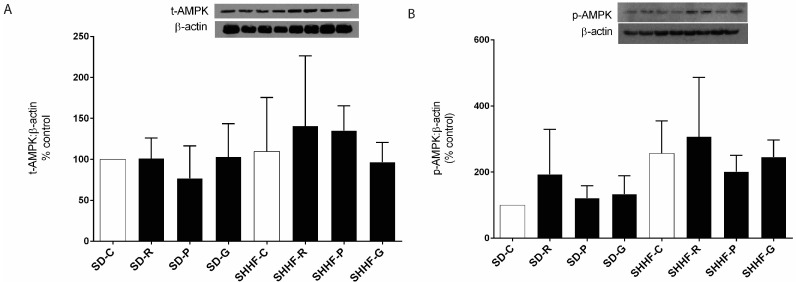
Lack of effect of stilbenoid treatment on AMPK in SD and SHHF rat hearts. No differences in (**A**) total AMPK levels nor (**B**) AMPKα activation (vis-à-vis phosphorylation at Thr172) were detected between SD and SHHF rat hearts, in the presence or absence of stilbenoid treatment. *n* = 5.

**Figure 7:**
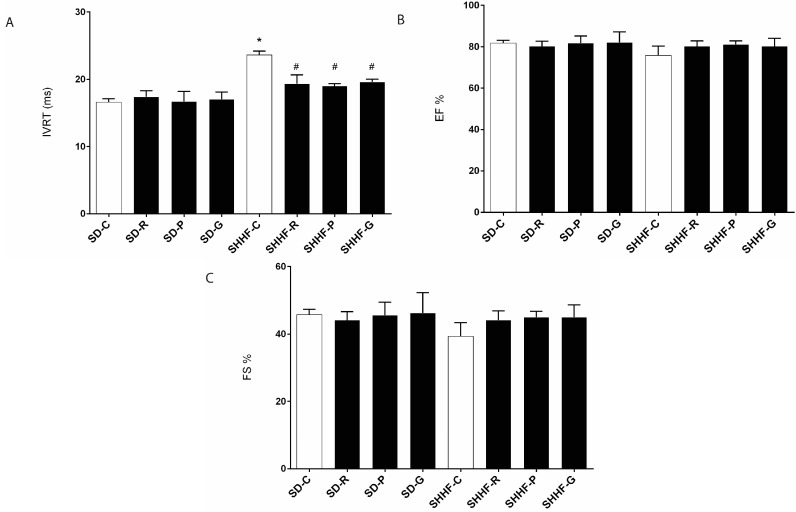
Stilbenoid treatment improves diastolic function in the SHHF rat. Compared to hearts from untreated SD rats (SD-C), (**A**) IVRT was impaired (i.e., prolonged) in SHHF rats, whereas parameters of systolic function such as (**B**) ejection fraction (EF) and (**C**) fractional shortening remained normal. 8-week treatment with resveratrol (R; 2.5 mg/kg/day), pterostilbene (P; 2.5 mg/kg/day), or gnetol (G; 2.5 mg/kg/day) improved IVRT. *n =* 6–8. * *p* < 0.05 vs. SD-C hearts; ; ^#^ *p* < 0.05 vs. SHHF-C.

Systolic, diastolic and mean blood pressures were elevated in SHHF rats compared with SD rats (Table 1). Early cardiac hypertrophy was also evident in SHHF rats, as normalized intraventricular septal thickness (end diastole: IVSd and end systole: IVSs) were augmented compared with age-matched SD rats (Figure 5), and this is an indicator of left ventricular hypertrophy. Asymmetrical interventricular septal hypertrophy (ASH) is also a predominant diagnostic feature of hypertrophic cardiomyopathy (HCM), which is commonly characterized by the presence of disproportionately hypertrophied septum in HCM patients. Interestingly, hypertensive patients may also develop ASH along with an increase in left ventricular mass [39,40]. AMPK levels and activation status were unaffected in SHHF rats nor by stilbenoids (Figure 6). Left ventricular isovolumic relaxation time (IVRT), an indicator of diastolic function, was impaired in SHHF rats, whereas systolic function (i.e., ejection fraction and fractional shortening) was normal (Figure 7). Although stilbenoid treatment had no effect on hypertrophy in vivo (Figure 5) nor AMPK (Figure 6), resveratrol, gnetol, and pterostilbene significantly improved IVRT (Figure 7).

## 3. Discussion

To our knowledge, the present study shows for the first time that the resveratrol analogs pterostilbene and gnetol, at least in part, attenuate hypertrophy of isolated cardiomyocytes via AMPK signaling. This is consistent with previous reports first, of the ability of resveratrol to prevent cardiomyocyte hypertrophy in vitro, in response to stimuli such as angiotensin II [28,29], phenylephrine [30], and norepinephrine [31], and second, of the anti-hypertrophic role of AMPK [29,31]. Therefore, we continued to evaluate the ability of resveratrol, pterostilbene, and gnetol to suppress cardiac hypertrophy in the SHHF rat. The SHHF rat models human heart disease in that it develops hypertension and hypertrophy that progress to decompensated heart failure [41,42]. To our knowledge, this is the first study of resveratrol (and structural analogs) in the SHHF rat.

We hypothesized that, because pterostilbene exhibits improved bioavailability and prolonged half-life compared to resveratrol [14,18], we would observe greater anti-hypertrophic actions with pterostilbene (and perhaps gnetol) in vivo. Despite the anti-hypertrophic effects that we observed in isolated cardiomyocytes, and the ability of resveratrol to attenuate hypertrophy in aortic-banded rats [25,26] and SHR [11,27] in vivo, stilbenoid treatment failed to attenuate left ventricular hypertrophy in SHHF rats.

There are a number of possible explanations for this discrepancy, and a major candidate we suggest is strain differences. The SHHF rat models genetic predisposition to heart failure superimposed on hypertension, whereas SHR models hypertension alone [43]. Therefore, despite the fact that we selected a dose of 2.5 mg/kg/day based on our previous findings that this dose of resveratrol suppressed hypertrophy in aortic-banded rats and SHR [11,25,26], SHHF rats and SHR may respond differently to pharmacotherapy. A similar notion was previously reported by Sharkey et al. where, for example, SHHF rats exhibited cardiac sensitivity to doxorubicin that was attenuated compared to that of SHR or normotensive WKY rats [43]; the authors likewise suggested strain differences in arachidonic acid metabolism. It is plausible, therefore, that equivalent doses of resveratrol would act differentially in hearts from SHHF rats.

In fact, our findings suggest that the strain differences contributing to differential resveratrol responsiveness in SHHF rats vs. SHR may relate to AMPK. We observed gnetol- and pterostilbene-dependent AMPK activation in isolated cardiomyocytes, and this is consistent with the ability of resveratrol to activate AMPK in SHR hearts [27,31]. However, in contrast to SHR [27], we found that AMPK signaling is not impaired in SHHF rat hearts, and neither resveratrol, pterostilbene, nor gnetol activated AMPK in the hearts of SHHF rats. It also bears mentioning that activated AMPK may not exert anti-hypertrophic effects in SHHF rats anyhow. Cittadini et al. reported that metformin induced significant AMPK activation in SHHF rats, and yet failed to attenuate cardiac hypertrophy measured as normalized heart weights and cardiomyocyte diameters [44]. Nonetheless, our findings highlight the distinction between SHR and SHHF rats as different genetic models of cardiovascular disease; in fact, pathophysiological complexity may be augmented in the SHHF rat, given the combination of hypertension and heart failure predisposition. Thus, comparative effects of these stilbenoids remain to be determined in another experimental model such as SHR.

It is difficult to ascertain precisely why gnetol only transiently increased phosphorylation of AMPK (Figure 2). We speculate that this may be related first, to its half-life following oral administration (100 mg/kg; ~4.20 h) [45] and therefore second, at least in part, to the possible trend of increased total AMPK at 4 h that seems to decline similarly to p-AMPK at 8 and 24 h (Figure 2A,B). Importantly, the transient nature of AMPK activation is not inconsistent with the mechanistic role of AMPK that we propose. In a separate study on the anti-hypertrophic actions of endocannabinoids, we reported that phosphorylative activation of AMPK peaked by 4 h, and returned to baseline by 24 h [46]. This suggests that AMPK activation might occur at a key, initial time point that triggers signaling consequences that would in turn attenuate cardiomyocyte hypertrophy. For example, disruption of RhoA/RhoA kinase (ROCK) might be one such downstream consequence. These Rho GTPases are early effectors of hypertrophic growth [47,48,49]. In addition, AMPK crosstalk with eNOS might confer anti-hypertrophic effects [31,46,50,51], and NO inhibits ET1-induced cardiomyocyte hypertrophy, at least in part, by blocking the RhoA/ROCK cascade [52]. We therefore speculate that stilbenoids, by transiently activating AMPK, elicit NO-dependent blockade of RhoA/ROCK; the effects of stilbenoids such as gnetol and resveratrol on RhoA/ROCK signaling remain to be determined.

Our data show that untreated SHHF exhibited prolonged IVRT, and that this was restored towards normal by all stilbenoid treatments. First, this finding serves as indirect evidence that the stilbenoid compounds, and/or perhaps their bioactive metabolites [27], were indeed reaching the heart to produce equivalent in vivo effects. The purportedly improved oral bioavailability of pterostilbene [14,18] did not influence its effects on IVRT compared to resveratrol or gnetol. Second, prolonged IVRT reflects impaired myocardial relaxation and is therefore an indicator of diastolic dysfunction. This is the first demonstration of improved diastolic function in the SHHF rat with stilbenoid treatment. In contrast, ejection fraction and fractional shortening, both parameters of systolic function, were not yet significantly impaired in SHHF rats; this is consistent with previous reports that systolic dysfunction doesn’t emerge until at least 9 months of age, and was detected in 15-month old rats [41]. As fibrosis is a major contributor to IVRT prolongation, it is plausible that stilbenoid treatment attenuated interstitial remodeling. Indeed, resveratrol reportedly inhibits proliferation and differentiation of cardiac fibroblasts in vitro [53]. Accordingly, resveratrol attenuated fibrosis in vivo in a number of models including the fructose-fed rat [54], angiotensin II-treated mouse [55], DOCA-salt rat [56], and SHR [57]. The ability of resveratrol to attenuate cardiac fibrosis in SHR was associated with alleviation of oxidative stress and inflammation [57], so it is plausible that resveratrol, pterostilbene, and gnetol acted similarly in SHHF rats, though this remains to be determined.

In conclusion, we observed equivalent effects in vitro (i.e., anti-hypertrophic) and in vivo (i.e., improved diastolic function). However, this study highlights the possibility of differential responses to stilbenoid polyphenols between in vitro models and distinct in vivo models (i.e., SHR vs. SHHF rats). This is an important consideration when pursuing stilbenoid-based therapies for human cardiovascular disease with its complex multi-factorial pathophysiology.

## 4. Materials and Methods

### 4.1. General Information

Endothelin-1, resveratrol, pterostilbene, compound C, α-actinin antibody, Alexa Fluor goat anti-mouse secondary antibody, β-actin antibody, and polybrene were from Sigma-Aldrich (Oakville, ON, Canada). Triton X-100 was from EMD Millipore (Billerica, MA, USA). DMEM was from ThermoFisher Scientific (Missisauga, ON, Canada). AMPK and phosphorylated AMPK antibodies were from Cell Signaling Technology (Whitby, ON, Canada). Click-iT^®^ AHA Alexa Fluor^®^ 488 Protein Synthesis HCS Assay kit and calcein-AM were from Molecular Probes/Invitrogen (Burlington, ON, Canada). Inverted fluorescent microscope (Olympus 1X81, Markham, ON, Canada), Medical film processor (Konica SRX-101A, Taiwan), Electrophoresis power supply (Biorad Powerpac Basic, Mississauga, ON, Canada), Microplate reader (Fluostar Omega, BMG Labtech, Offenburg, Germany), Incubator (ThermoForma direct heat CO_2_ incubator).

### 4.2. Gnetol Synthesis Preparation of (E)-2,3’,5’,6-Tetramethoxystilbene (Scheme 1 *(**3**)*)

**Scheme 1:**
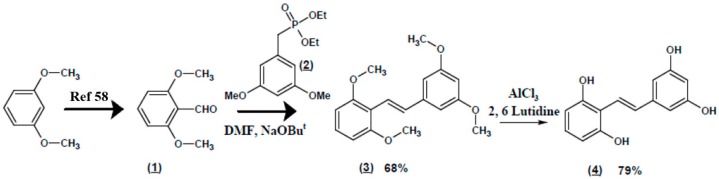
Synthesis of gnetol.

Diethyl 3,5-dimethoxybenzylphosphonate (Scheme 1, (**2**)) (144.6 g of 90% assay, 0.45 mol) was placed in a 500 mL four-necked round bottomed flask fitted with a mechanical stirrer, an addition funnel and a thermometer under dry nitrogen atmosphere. Dry dimethylformamide (140 mL) was added to this compound with stirring for dissolution. Sodium tert-butoxide (65 g, 0.68 mol) was added slowly to this reaction mixture at ambient temperature with stirring. The reaction mixture was cooled to 0 °C after 45 min and 2,6-dimethoxybenzaldehyde (Scheme 1, (**1**), 75 g, 0.45 mol) dissolved in dry dimethylformamide (40 mL) was dropped into the reaction mixture at 0–5 °C through an addition funnel. Upon completion of the addition, the reaction mixture was allowed to warm to ambient temperature spontaneously with stirring overnight. H_2_O (500 mL) was added to the reaction mixture slowly with stirring at ambient temperature and the mixture acidified with 1:1 H_2_O:hydrochloric acid (140 mL). The precipitate was filtered by suction, washed with H_2_O until neutral and dried in vacuo at 70 °C to obtain the pure product (*E*)-2,3’,5’,6-tetramethoxystilbene (Scheme 1, (**3**)) (92 g, 68% yield) as a pale yellow powder, m.p. 92–93 °C. HPLC purity of this compound is 99.2%.

^1^H-NMR (CDCl_3_, 300 MHz): δ 3.82 (s, 6H, 3’ & 5’-OC**H_3_**), 3.88 (s, 6H, 2- & 6- OC**H_3_**), 6.36 (t, *J* = 2.4 Hz, 1H, C_4’_-**H**), 6.58 (d, *J* = 8.4 Hz, 2H, C_3_-**H** & C_5_-**H**), 6.70 (d, *J* = 2.4 Hz, 2H, C_2’_-**H** & C_6’_-**H** ), 7.16 (t, *J* = 8.4 Hz, 1H, C_4_-**H** ), 7.46 (AB q, *J* = 16.2 Hz, 2H, trans ethene H). See Figure S2.

^13^C-NMR (CDCl_3_, 75 MHz): δ 55.56 (C_3’_- & C_5’_-O**C**H_3_), 56.00 (C_2_- & C_6_-O**C**H_3_), 99.56 (**C**_4’_-), 104.18 (**C**_2’_- & **C**_6’_-) 104.69 (**C**_3_- & **C**_5_-), 114.77 (**C**_1_-), 120.67 (Ethene C), 128.43 (**C**_4_-), 132.48 (Ethene C), 141.40 (**C**_1’_-), 158.87 (**C**_2_- & **C**_6_-), 161.02 (**C**_3’_- & **C**_5’_-). See Figure S3.

LC-MS: Positive APCI *m/e* 301 (M^+^+H).

#### Preparation of Gnetol (Scheme 1, (**4**))

2,6-Lutidine (142.6 g, 1.33 mol) was added to a 500 mL four-necked round bottomed flask fitted with a mechanical stirrer, an addition funnel and a thermometer under dry nitrogen atmosphere. Toluene (175 mL) was mixed with 2,6-lutidine with stirring. Anhydrous aluminum chloride (178.0 g, 1.33 mol) was slowly added to this solution of 2,6-lutidine in toluene at such a rate that the temperature did not rise above 50 °C. Upon completion of the addition, the temperature spontaneously increased to 50 °C. (*E*)-2,3’,5’,6-Tetramethoxystilbene (Scheme 1, (**3**)) (50 g, 0.17 mol) dissolved in toluene (175 mL) was added to the reaction mixture through the addition funnel, and the mixture slowly heated to 80 °C. This reaction mixture was stirred at 80 °C for 1 h and then slowly poured over ice (300 g). The quenched reaction mixture was acidified with concentrated hydrochloric acid (150 mL) and extracted with ethyl acetate (3 × 500 mL). The ethyl acetate layer was dried over anhydrous sodium sulfate (200 g), filtered and the solvents stripped off by a rotary evaporator to obtain crude gnetol (Scheme 1, (**4**)) (39 g). The crude gnetol (Scheme 1 (**4**)) was dissolved in acetone (100 mL) and passed through a column of neutral alumina (100 g). The absorbed product on neutral alumina was eluted with acetone (200 mL). The acetone eluents combined, the solvent was distilled off by a rotary evaporator and the residue was dried in vacuo at 80 °C. The dry residue was then triturated with dichloromethane (100 mL) followed by 2% methanol in dichloromethane (100 mL). The filtered solid residue was dried in vacuo at 80 °C to obtain pure gnetol (Scheme 1 (**4**)) (32 g, 79% yield) as a grey colored powder, m.p. 231.8–232.9 °C. HPLC purity of this compound is 99.5%.

^1^H-NMR (DMSO-*d*_6_, 300 MHz): δ 6.07 (t, *J* = 2.1 Hz, 1H, C_4’_-**H**), 6.32 (d, *J* = 2.1 Hz, 2H, C_2’_-**H** & C_6’_-**H**), 6.33 (d, *J* = 8.1 Hz, 2H, C_3_-**H** & C_5_-**H**), 6.82 (t, *J* = 8.1 Hz, 1H, C_4_-**H**), 7.30 (AB q, *J* = 16.2 Hz, 2H, trans ethene H), 9.16 (s, 2H, -OH), 9.64 (s, 2H, -OH). See Figure S4.

^13^C-NMR (DMSO-*d*_6_, 75 MHz): δ 101.42 (**C**_4’_-), 103.94 (**C**_2’_- & **C**_6’_-), 106.64 (**C**_3_- & **C**_5_-), 111.19 (**C**_1_-), 120.47 (Ethene C), 127.88 (**C**_4_-), 130.15 (Ethene C), 140.93 (**C**_1’_-), 156.80 (**C**_2_- & **C**_6_-), 158.55 (**C**_3’_- & **C**_5’_-). See Figure S5.

LC-MS: Negative ESI *m/e* 243 (M − H)**^−^**.

In summary, 2,6-Dimethoxybenzene was converted to 2,6-dimethoxybenzaldehyde (**1**) through a lithiation-formylation sequence as described in the literature [58]. As depicted in Scheme 1, compound **1** was used in the subsequent Emmons-Horner coupling with diethyl 3,5-dimethoxy- benzoylphosphonate (**2**) to yield exclusively the *trans*-isomer, (*E*)-2,3′,5′,6-tetramethoxystilbene (**3**). The *trans*-geometry purity was evident from the NMR coupling constant data for the olefinic protons (*J* = 16.2 Hz) (Figures S2 and S3, Supplementary Materials). Tetra-demethylation of **3** was effected by the aluminum chloride/lutidine complex, which is shown as an example. This may be achieved by other amines such as *N*,*N*-diethylaniline [59]. We believe that the polymeric nature of aluminum chloride is disrupted by the amine substrate to yield a more powerful Lewis acid that catalyzes the tetra-*O*-demethylation reactions efficiently. The product gnetol (**4**) was isolated again as a pure *trans*-isomer as evidenced by NMR coupling constant data for the olefinic protons (*J* = 16.2 Hz) (Figures S4 and S5, Supplementary Materials). The scheme was easily applicable to large scale synthesis.

### 4.3. Animals

This study was approved by the University of Manitoba Animal Care Committee and follows Canadian Council of Animal Care guidelines.

### 4.4. In Vitro Experiments

#### 4.4.1. Neonatal Rat Ventricular Myocytes

Ventricular myocytes were isolated from 1-day-old neonatal Sprague-Dawley rats by digestion with several cycles of 0.1% trypsin and mechanical disruption, as previously described [60]. Cells were cultured on gelatin-coated plates in DMEM containing 10% cosmic calf serum (CCS; GE Healthcare Life Sciences, South Logan, UT, USA) for 18–24 h prior to experimentation.

#### 4.4.2. Treatments

As applicable, myocytes were subjected to lentiviral infection. Myocytes were then rendered quiescent by serum deprivation for 24 h and pretreated with gnetol (1–100 µg/mL) or pterostilbene (1–50 µg/mL) in the presence or absence of a chemical inhibitor of AMPK (compound C [i.e., 6-[4,[4-(2-piperidin-1-ylethoxy)phenyl]-3-pyridin-4-ylpyrazolo[1,5,[1,5-a]pyrimidine]; 1 μM; 1 h). Following the 1 h pretreatment, ligands remained in the culture media for the remainder of the experiment. Hypertrophy was stimulated by addition of ET1 (0.1 µM; 24 h).

#### 4.4.3. Hypertrophic Indicators

Hypertrophy was assessed as previously described [61,62]. Briefly, myocyte size was assessed by immunofluorescence, fluorescence microscopy, and computer-assisted planimetry. De novo protein synthesis was measured using the Click-iT^®^ AHA Alexa Fluor^®^ 488 Protein Synthesis HCS Assay kit (Invitrogen), according to the manufacturer’s protocol.

#### 4.4.4. Measurement of Cardiomyocyte Viability

Cardiomyocyte viability was assessed by staining with calcein-AM. Calcein AM is a membrane-permeant dye that is converted to a green-fluorescent calcein by intracellular esterases in viable cells. Following treatments and removal of media, 3 µM calcein-AM in warm PBS was added to each well (24-well plate, 350,000 cells/well). The following were used as controls: background − no cells + calcein AM; live controls − vehicle-treated cells; dead cell controls − cells treated with 0.2% Triton X-100 (15 min). Following dark incubation (37 °C, 30 min), fluorescence was measured using a plate reader using excitation/emission wavelengths 485 nm/535 nm for calcein.

#### 4.4.5. Western Blotting

Cell or tissue lysates were prepared in RIPA buffer, clarified by centrifugation, and p-AMPK and AMPK were detected by conventional western blotting. Membranes were stripped and reprobed with β-actin antibody to account for loading variations among lanes.

#### 4.4.6. Lentiviral Preparation and Infection

Lentiviral vectors expressing shRNA against AMPK α_1_ and α_2_ were obtained from the University of Manitoba Open Biosystems library (AMPK α_1_: TRCN0000000860, TRCN0000024003; AMPK α_2_: V2LMM_73754, V2LMM_71195). Scrambled sequences served as non-silencing controls. Lentivirus vector plasmids were co-transfected with psPAX2 (packaging) and pMD2.G (enveloping) vectors using FuGENE6 Reagent (Roche; Indianapolis, IN, USA). High-titer lentiviral stock was produced in HEK-293T cells 48 h after transfection. Myocytes were infected for 24 h by application of the lentivirus to the culture medium. Knockdown was confirmed by western blotting (data not shown).

### 4.5. In Vivo Experiments 

#### 4.5.1. Experimental Animals

Male Sprague Dawley (SD) rats and lean spontaneously hypertensive heart failure (SHHF) rats were obtained from Charles River (Senneville, QC, Canada) at 7 weeks of age. Animals were housed under a 12-h light/dark cycle at 22 °C and 60% humidity and fed *ad libitum*.

Rats were trained for blood pressure measurement using tail cuff plethysmography (CODA non-invasive blood pressure system; Kent Scientific, Torrington, CT, USA), after 14 days of acclimatization. SD and SHHF rats were treated for 8 weeks by oral gavage with vehicle or resveratrol, pterostilbene, and gnetol (2.5 mg/kg/day; Sigma Aldrich-Canada; Cayman Chemical, Ann Arbor, MI, USA; and Sabinsa Corporation, East Windsor, NJ, USA). This dose was chosen based on our previous study that showed vascular improvement using low dose resveratrol in the SHR animal model [10].

#### 4.5.2. Echocardiography

Rats were anesthetized with isoflurane (induction: 3%; maintenance: 2%), and transthoracic echocardiography was performed via 2D guided M-mode and Doppler imaging modalities with a 13-MHz probe (Vivid E9; GE Medical Systems, Milwaukee, WI, USA). The 2D parasternal, short-axis view was used to image the heart at the papillary muscle level. Doppler flow velocity tracings were obtained at the level of the mitral valve in the apical four-chamber view and at the level of aorta in the five chamber view, with the Doppler probe placed at the edge of the mitral leaflets and aortic valve, respectively. All measurements were performed, according to the recommendations of the American Society for Echocardiography leading-edge method, from three consecutive cardiac cycles using EchoPAC software (GE Medical Systems). Systolic functional parameters such as percentage of left ventricular ejection fraction (EF) and fractional shortening (FS) were determined from parasternal short-axis view image-based end-systolic and end-diastolic diameters and volumes. Diastolic functional parameters such as isovolumic relaxation time (IRVT) was obtained from Doppler tracing. Morphological measurements such as interventricular septal wall thickness (IVS) at diastole and systole were determined from the parasternal short-axis view images [11].

### 4.6. Statistics

Data are presented as means ± standard deviation with the exception of controls. Replicates were generated as separate experiments from distinct myocyte preparations. As each *n* = 1 represents one specific untreated group serving as the control for one specific treated group, and the normalization is achieved on a 1 by 1 basis (in essence, pairing groups), errors could not be validly derived for the control groups which were set at 100%. All data were subjected to 1-way ANOVA followed by a Newman-Keuls Multiple Comparison test to detect between-group differences. *p* < 0.05 was considered significant.

## Figures and Tables

**Table 1:** Effect of stilbenoid polyphenols on blood pressure.

Parameter (mm Hg)	SD				SHHF			
	C	R	P	G	C	R	P	G
**Systolic BP**	140 ± 18	130± 18	140 ± 22	136 ± 16	195± 10 ^a^	187 ± 14 ^a^	192 ± 14 ^a^	207 ± 10 ^a^
**Diastolic BP**	98 ± 14	85 ± 20	100 ± 21	94 ± 14	143 ± 7 ^a^	137 ± 16 ^a^	135 ± 20 ^a^	152 ± 7 ^a^
**Mean BP**	112 ± 16	100 ± 19	113 ± 21	108 ± 14	160 ± 8 ^a^	153 ± 15 ^a^	154 ± 18 ^a^	170 ± 8 ^a^
**Pulse Pressure**	43 ± 5	46 ± 5	41 ± 5	42 ± 4	52 ± 6	50 ± 7	56 ± 9 ^a^	54 ± 4 ^a^

C—control, R—resveratrol, P—pterostilbene, G—gnetol. ^a^ *p* < 0.05 vs. SD controls.

## Supplementary Materials

Supplementary materials can be accessed at: http://www.mdpi.com/1420-3049/22/2/204/s1.
