# Supplementary material for: Disparate Effects of Stilbenoid Polyphenols on Hypertrophic Cardiomyocytes In Vitro vs. in the Spontaneously Hypertensive Heart Failure Rat
Source: Molecules. 2017 Feb 1;22(2):204. doi: 10.3390/molecules22020204 (PMC6155878; doi:10.3390/molecules22020204)
Supplement: Supplementary file 1 [file molecules-22-00204-s001.pdf]

Supplementary Materials: Disparate Effects of Stilbenoid Polyphenols on Hypertrophic Cardiomyocytes In Vitro vs. in the Spontaneously Hypertensive Heart Failure Rat

Bolanle C. Akinwumi, Pema Raj, Danielle I. Lee, Crystal Acosta, Liping Yu, Samuel M. Thomas, Kalyanam Nagabhushanam, Muhammed Majeed, Neal M. Davies, Thomas Netticadan and Hope D. Anderson

Resveratrol Prevents ET1-Induced Cardiomyocyte Enlargement

Myocytes were rendered quiescent by serum deprivation for 24 h and pretreated with resveratrol (1–100 µg/mL). Following the 1 h pretreatment, resveratrol remained in the culture media for the remainder of the experiment. Hypertrophy was stimulated by addition of ET1 (0.1 µM; 24 h; Figure S1). Lower concentrations of resveratrol (1–10 µg/mL) abolished ET1-induced myocyte enlargement, but did not affect untreated myocytes. In contrast, higher concentrations of resveratrol (50–100 µg/mL) markedly reduced cell size in the presence and absence of ET1, suggesting toxicity.

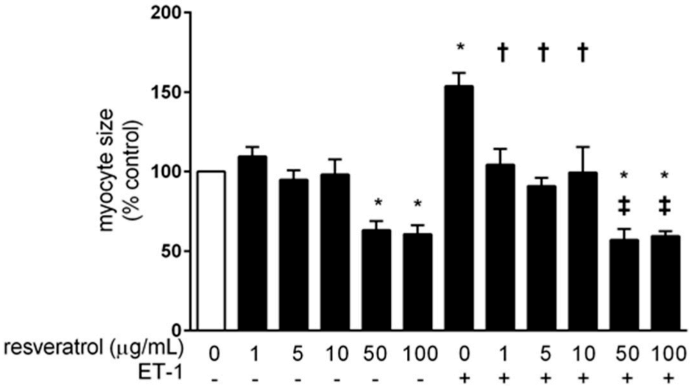

Figure S1. Resveratrol attenuates ET-1-induced cardiomyocyte hypertrophy. * $p < 0.05$ and ** $p < 0.01$ vs. control (open bars); † $p < 0.05$ and ‡ $p < 0.01$ vs. ET1.

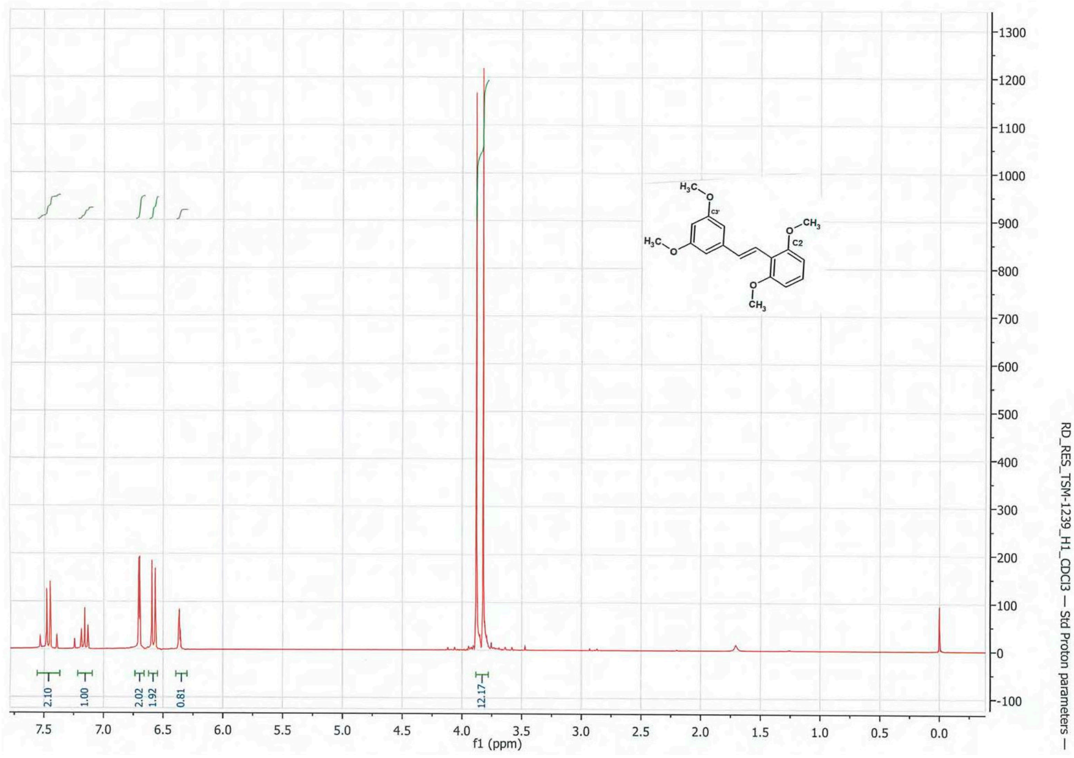

Figure S2. ^{1}H-NMR of tetramethoxystilbene.

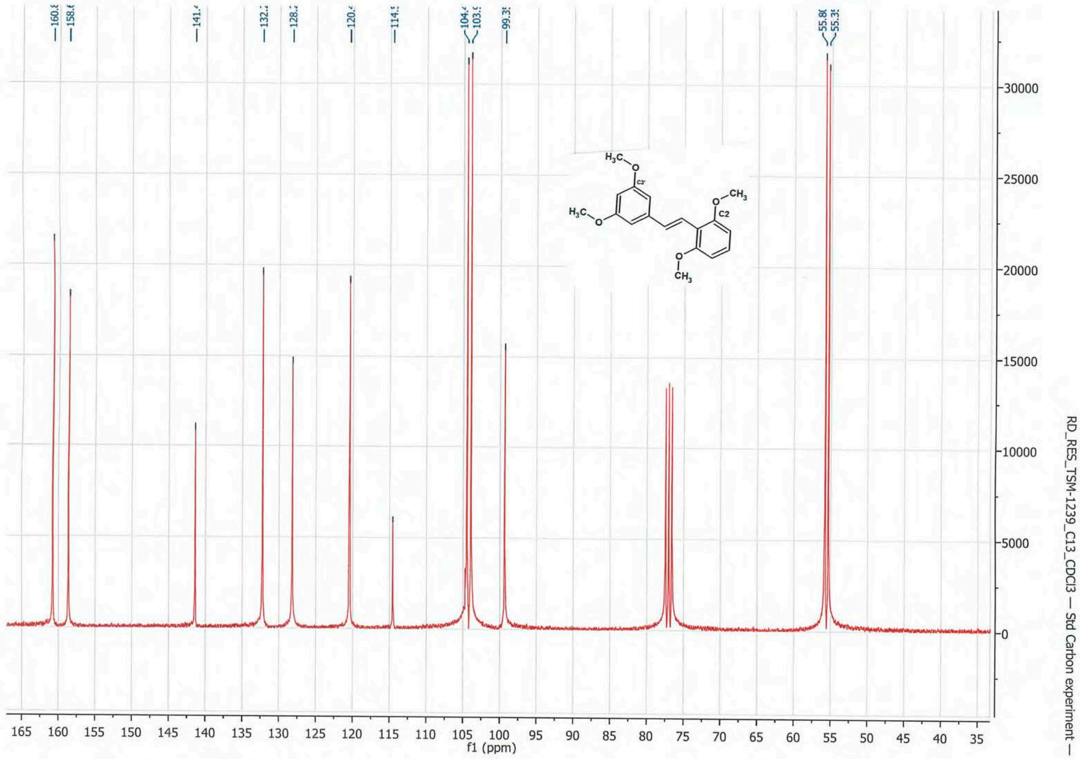

Figure S3. ^{13}C-NMR of tetramethoxystilbene.

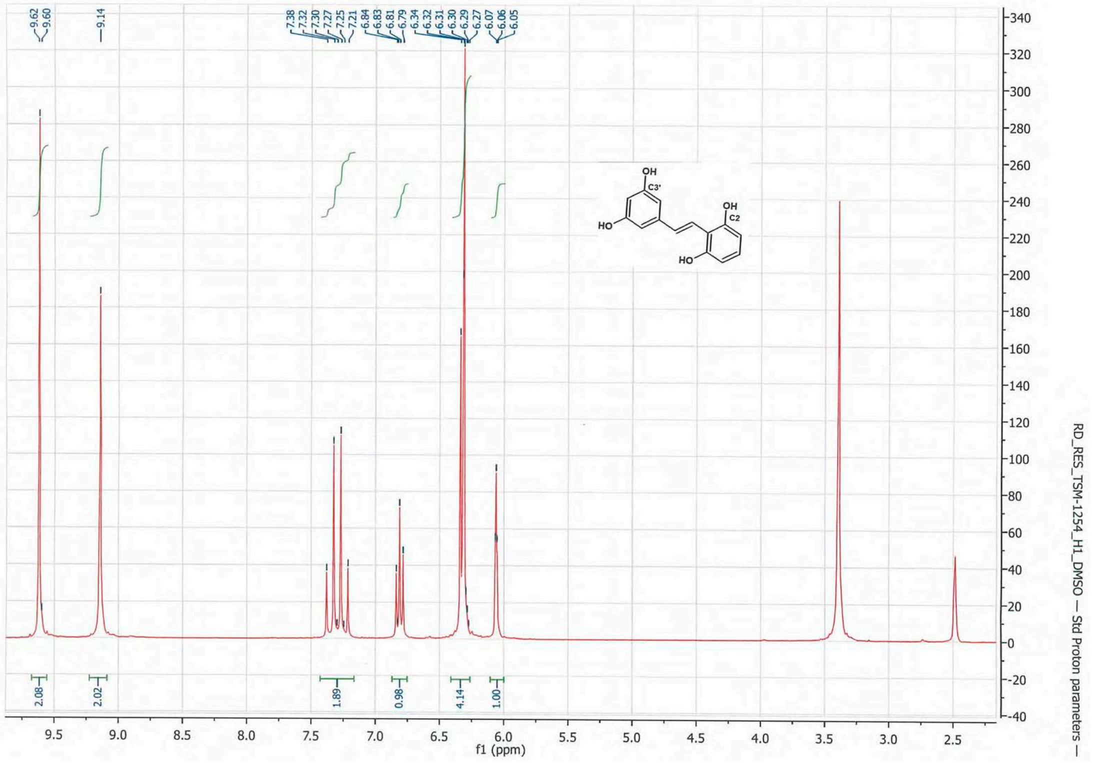

Figure S4. ^{1}H-NMR of gnetol.

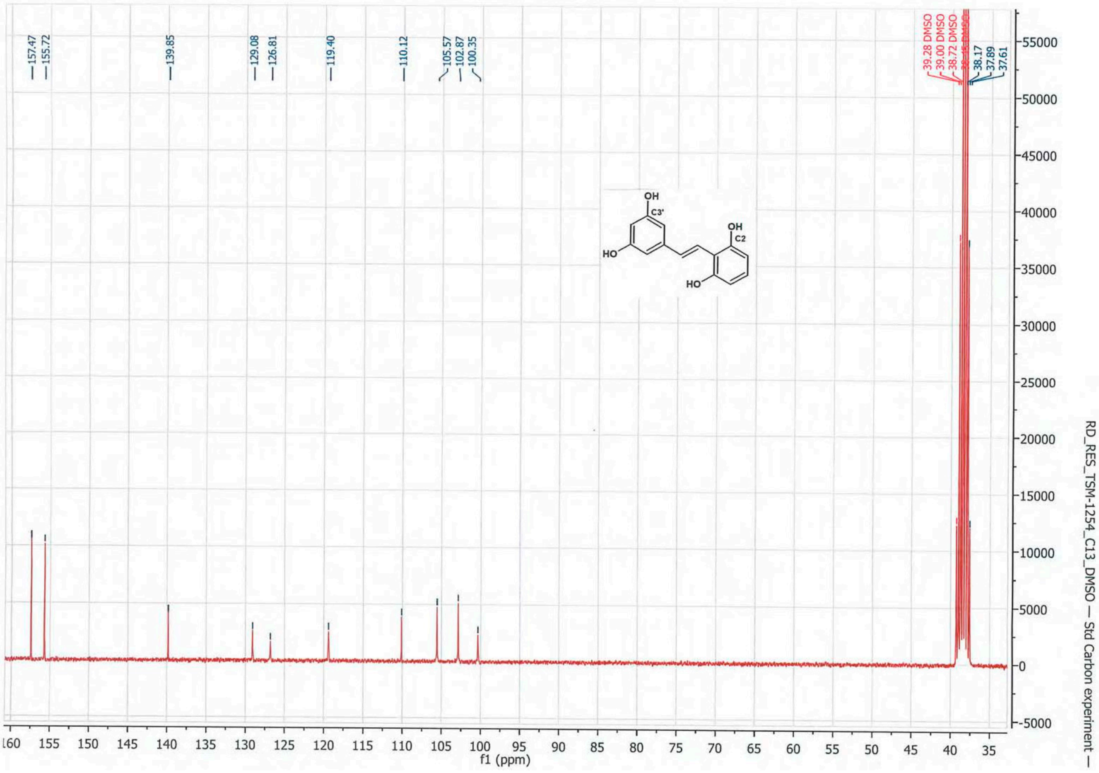

Figure S5. ^{13}C-NMR of gnetol.
